# Heart–Liver Interplay in Patients with Fontan Circulation

**DOI:** 10.3390/jcm14041114

**Published:** 2025-02-09

**Authors:** Roberta Biffanti, Jolanda Sabatino, Alice Pozza, Liliana Chemello, Luisa Cavalletto, Andrea Gasperetti, Massimo Padalino, Giovanni Di Salvo

**Affiliations:** 1 Paediatric Cardiology Unit, Department of Woman’s and Child’s Health, University of Padua Medical School, 35121 Padova, Italy; 2 Paediatric Cardiology Unit, Magna Graecia University, 88100 Catanzaro, Italy; 3 PhD School in Developmental Medicine and Health Planning Sciences, University of Padua, 35122 Padua, Italy; 4 Internal Medicine & Hepatology Unit, Department of Medicine, University of Padua Medical School, 35121 Padova, Italy; liliana.chemello@unipd.it (L.C.); luisa.cavalletto@unipd.it (L.C.); 5 Sport and Exercise Medicine Unit, Department of Medicine, University of Padua Medical School, 35121 Padova, Italy; andrea.gasperetti@aopd.veneto.it; 6 Paediatric and Congenital Cardiac Surgery Unit, Department of Cardiac, Thoracic & Vascular Sciences and Public Health, University of Padua Medical School, 35121 Padova, Italy; 7 Section of Cardiac Surgery, Department of Precision and Regenerative Medicine, University of Bari “Aldo Moro”, 70121 Bari, Italy

**Keywords:** Fontan circulation, systemic atrioventricular valve, hepatic fibrosis, multidisciplinary follow-up

## Abstract

**Background:** The Fontan procedure has provided pediatric patients suffering from severe congenital heart disease the opportunity to reach adulthood. Increasingly, we encounter the liver repercussions of Fontan circulation, alongside a decline in heart function and exercise performance. This study aims to identify the univentricular heart malformations that are most susceptible to liver dysfunction; assess which markers of liver injury are essential for multidisciplinary clinical follow-up of Fontan patients; determine the optimal approach for evaluating liver function in Fontan patients; and explore how a congenital cardiology team can interpret the data and respond effectively to signs of organ failure. **Methods:** Cross-sectional clinical study including patients who underwent a Fontan procedure at the University Hospital of Padua between 1982 and 2017. Patients were admitted for elective hospitalization between June 2021 and June 2022 and underwent clinical assessment, laboratory tests, and instrumental examinations. **Results**: Seventy patients were included in the study. On admission, 48 patients (72%) were in New York Heart Association (NYHA) functional class I, and the cardiopulmonary exercise test was normal for age and gender. At laboratory tests, 56% of patients showed changes in NTproBNP values, most of whom had right-sided ventricular morphology. Liver function tests showed abnormal Gamma-Glutamyl Transferase (GGT) blood levels in 68%. On cardiac imaging, at least moderate atrioventricular valve insufficiency was found in 9% of cases. Fibroscan showed altered hepatic stiffness values in 25% of cases. Statistical analysis showed that systemic atrioventricular valve (SAVV) dysfunction was significantly associated with a reduction of maximum oxygen consumption (VO_2_ max) and hepatic stiffness. **Conclusions:** SAVV dysfunction is significantly responsible for worse functional outcomes and the development of hepatic fibrosis due to an increase in venous congestion. Setting up a careful multidisciplinary follow-up in these patients is mandatory for early detection of complications, prompt treatment, and better outcomes.

## 1. Introduction

The occurrence of functional single ventricle disease in newborns varies, with rates between 0.08 and 0.4 out of every 1000 births, and the Fontan procedure is commonly the chosen surgical palliation for these patients [1,2]. Originally performed in 1968, the Fontan operation has evolved significantly to enhance the circuit’s hydrodynamics, now typically involving a total cavo-pulmonary connection through an intra- or extra-cardiac conduit [3]. Although the procedure can achieve nearly normal arterial saturation and relieve chronic cardiac volume overload, the probability of remaining free from long-term morbidity is relatively low [4,5]. Advances in cardiac imaging techniques and improvement in the anatomical design of the Fontan circuit with a deeper grasp of its pathophysiology have lessened the risks of heart failure, thromboembolism, and arrhythmias [6,7]. Nonetheless, the high systemic venous pressure required to propel blood through the pulmonary system imposes stress on the abdominal organs, leading to liver disease and in extreme cases to protein-losing enteropathy (PLE) [8,9]. The phenomenon known as “Fontan-associated liver disease” (FALD) is a growing concern, potentially increasing morbidity and mortality in adulthood [10,11,12,13].

This study aims to (1) identify the univentricular heart malformations that are most susceptible to liver dysfunction; (2) assess which markers of liver injury are essential for multidisciplinary clinical follow-up of Fontan patients; (3) determine the optimal approach for evaluating liver function in Fontan patients; and (4) explore how the congenital cardiology team can interpret the data and respond effectively to signs of organ failure [7,14].

## 2. Material and Methods

### 2.1. Study Population

This is a clinical cross-sectional study including patients who underwent a Fontan procedure between 1982 and 2017 at the Department of Women’s and Children’s Health of the Padua University Hospital. Patients excluded from the study were those with unknown survival status followed elsewhere or those with congenital liver abnormalities.

All patients included in the study underwent elective hospitalization at the Paediatric Cardiology Unit between June 2021 and June 2022, to participate in the annual follow-up as per local protocol for patients with Fontan circulation. All data were collected while maintaining confidentiality and were anonymized for statistical analysis. The study was submitted to the attention of the Research Centre Ethical Committee of the University of Padua.

Clinical data extracted from Galileo’s eHealth medical platform of the Padua University Hospital included age, body mass index, body surface area, NYHA functional class, blood pressure, heart rate, rest oxygen saturation, and time since surgery.

### 2.2. Biochemistry Assesment 

Fasting laboratory blood tests included complete blood count, creatinine, cystatin C, urea, electrolytes, transaminases, Lactate Dehydrogenase (LDH), alpha-fetroprotein, insulin, uric acid, Alkaline Phosphatase (ALP), Aspartate Aminotransferase (AST), GGT, ammonium, troponin I, NT-proBNP, protein profile, alpha2 macroglobulin, transferrin, ferritin, pseudo-acetylcholinesterase, total cholesterol, vitamin D, anti-HCV antibodies, HBsAg and anti-HBsAb. Alpha1-antitrypsin was measured on stool.

### 2.3. Assessment of Cardiac Performance

Echocardiographic examinations were performed by three experienced pediatric cardiologists using General Electric Vivid E9 ultrasound System (GE Healthcare, Chicago, IL, USA). Chamber quantification measurements were assessed according to the current European Association of Echocardiography guidelines [15].

SAVV regurgitation (SAVVR) or stenosis was assessed from the apical chamber view and defined as absent, mild, moderate, or severe.

E-wave velocity (cm/s), A-wave velocity (cm/s), E/A ratio, deceleration time (ms), E’-wave velocity (cm/s, measured at the free wall of the dominant ventricle), E/E’ ratio, and D-wave deceleration time were assessed as part of diastolic performance evaluation. Diastolic function was also evaluated after a leg lifting test, with legs being passively lifted by the clinician for one minute to unmask a latent diastolic disfunction.

The E/E’ value was considered pathological above 12. The degree of diastolic dysfunction was assessed according to Margossian et al. [16]. Some of the parameters mentioned were not evaluable variants of univentricular anatomy. When analyzed as a dichotomous variable, diastolic function was considered to be normal (grade 0) or having some degree of dysfunction (grade 1 to 3).

In patients with a left single ventricle, systolic function was evaluated using mitral annular plane systolic excursion (MAPSE) and ejection fraction (EF%) with the two-dimensional (2D) Simpson method. In those patients with a right single ventricle, the ventricular function was evaluated using tricuspid annular plane systolic excursion (TAPSE) and fractional area change (FAC%). A cut-off of 35% was considered for FAC, while a cut-off of 50% was set for EF. For the global longitudinal strain (GLS) evaluation of apical four, three, and two chambers views were acquired with a frame rate of 60–100 frames/s, and the best quality images were then transferred to an offline workstation (Echopac Version 202, GE Healthcare) to perform strain analysis.

If not recently performed (<12 months), patients underwent a cardiopulmonary exercise test (CPET) at the Sport Medicine Department of Padua University. CPET was performed during hospitalization or within a short time after discharge (<6 months). Patients with physical limitations (e.g., severe neurological impairment, outcomes of cerebrovascular or ischemic events) were excluded from CPET.

As regards the interpretation of the maximum oxygen consumption data (VO_2_ max) at the CPET, in consideration of the different ages of the included patients, the absolute value (measured in mL/kg/min) was compared to the predicted sedentary healthy individuals of the same age and gender. The predicted value, expressed in percentage, was then compared with that reported in the literature for subjects with univentricular circulation of the same age group [17,18]. The final evaluation was therefore expressed qualitatively (good functional capacity, normal, borderline, mildly and moderately reduced) or considered dichotomously when necessary (normal versus borderline or reduced).

### 2.4. Liver Assessment

During hospitalization, liver ultrasound elastometry (Fibroscan) was conducted using the FibroScan^®^ 502 device (Echosens, Paris, France). The choice between a specific probe “M” (medium) or “S” (small) depended on the patient’s chest diameter. This probe facilitated the measurement of hepatic stiffness, splenic stiffness, and hepatic steatosis. Collected data encompassed various parameters, including the measurement of hepatic vein diameter, liver dimensions, assessment of echogenicity, echo-structure, hepatic margin, and presence of caudate lobe hypertrophy. Lesion detection, spleen diameter measurement, inferior vena cava (IVC) diameter, portal vein diameter with portal flow velocity, and evaluation of collateral circulation were also performed. The clinical-instrumental hepatological evaluation relied on these parameters. Cut-off values for platelet count, AST, ALT, and GGT values were considered based on age and gender references. A normal alpha-fetoprotein value was defined as <7.4 ug/L. For liver stiffness interpretation, a cut-off value of 22 kPa was used considering the likely concomitant role of hepatic venous congestion and fibrosis in Fontan patients [13]. Regarding splenic stiffness, in the absence of specific cut-off values for FALD, criteria from the BAVENO VI consensus were adopted, along with a cut-off value > 46 kPa [19]. Splenic stiffness was also evaluated as a continuous variable to correlate with other parameters. Three risk assessment scores for advanced liver disease in adults with chronic non-FALD liver disease were applied:

- LSPS (liver stiffness-spleen size to platelet ratio)
- APRI (AST to platelet ratio index)
- FIB-4 (Fibrosis 4)

LSPS, validated in chronic liver disease, determines portal hypertension risk with esophageal varices, with cut-offs of 1.1 and 1.2 for esophageal varices and high-risk esophageal varices, respectively [20,21]. When applied to adult Fontan patients, it exhibited sensitivity and specificity of 91.3% and 75% for advanced FALD diagnosis (defined as splenic diameter > 120 mm, portal vein diameter > 12 mm, portal flow velocity < 12 cm/s, or presence of collateral circulation) with a cut-off of 1.31. APRI and FIB-4, used for fibrosis and cirrhosis risk evaluation in non-FALD patients, demonstrated lower sensitivity and specificity in evaluating advanced FALD in adult Fontan patients (APRI sensitivity 65.2%, specificity 65% with cut-off > 0.49; FIB-4 sensitivity 48%, specificity 95% with cut-off > 1.66) [22,23].

### 2.5. Statistical Analysis

The study design is a prospective cohort. The descriptive analysis of the study population was expressed as mean ± standard deviation for the continuous variables with normal distribution or as median and interquartile range for the quantitative variables with non-normal distribution; absolute frequency and percentage were used for discrete variables. The association between the continuous quantitative variables that demonstrated statistical significance and/or clinical relevance was expressed by the Pearson correlation index. As regards the qualitative variables, however, the groups were compared using the chi-square test (X2) or Fisher’s exact test, when appropriate. A *p*-value < 0.05 was considered statistically significant. All analyses were performed using SPSS Statistics software (version 28.0.0.0).

## 3. Results

### 3.1. General Population: Clinical Data

#### 3.1.1. Anatomical Findings

The study included 70 patients (M/F = 37/33), with a mean age of 21 ± 9 years at the time of hospital admission, ranging from 10 to 47 years; 32 (46%) were younger than 18 years. On average, 16.4 ± 8.5 years had passed since Fontan palliation, with a range spanning from 4.7 to 40 years. Treatment types varied: 49 patients (70%) received an extra-cardiac conduit, 15 (21%) had a lateral intra-cardiac tunnel, and 6 (9%) underwent a Björk atrio-infundibular anastomosis.

Looking at the specific cardiac lesions, 13 patients (19%) had hypoplastic left heart syndrome (HLHS), 11 (16%) were diagnosed with double-inlet left ventricle (DILV), and 8 (11%) with pulmonary atresia and intact interventricular septum (PA/IVS). Additionally, there were 15 patients (21%) with tricuspid atresia (TA), 5 (7%) with an unbalanced complete atrioventricular canal (CAVC-s), 7 (10%) with double-outlet right ventricle (DORV), 4 (6%) with mitral atresia (MA), 2 (3%) with criss-cross heart, and 5 (7%) presented with other anomalies. Ventricular morphology was classified as single right ventricle in 25 patients (36%), single left ventricle in 38 patients (54%), and indeterminate in 7 patients (10%).

#### 3.1.2. Functional Status

Of the 67 patients evaluated, 48 (72%) were in NYHA functional class I, while 19 (28%) were in class II or III, with a greater limitation in physical activity. Three patients could not be classified due to severe neuromotor impairment, and no patients were in class IV.

#### 3.1.3. Laboratory Exams

The average hemoglobin level was 15.3 mg/dL, indicating chronic cyanosis, with levels ranging from 9.4 to 18.4 mg/dL. NT-proBNP levels were elevated (>125 ng/L) in 37 out of 66 patients (55%); this group included 17 patients with a functional single left ventricle and 20 patients with a functional single right ventricle.

Table 1 reports the clinical data of our cohort, including demographics, surgical distribution of univentricular physiology, and relevant biochemical findings.

#### 3.1.4. Medication

Eighteen patients (26%) were on warfarin, 42 (61%) were taking acetylsalicylic acid, and 10 patients were not on any anticoagulation or antiplatelet therapy. Beta-blockers were being used by nine patients (13%), and twenty-two (32%) were on systemic vasodilators.

#### 3.1.5. Arrhythmias

A 24 h Holter monitor test was performed by 50 patients. Results showed that 39 patients (78%) had no arrhythmias. Six patients (12%) had minor tachyarrhythmias, such as supraventricular or ventricular ectopic beats, four (8%) had major tachyarrhythmias including atrial flutter and paroxysmal supraventricular tachycardia, and one (2%) exhibited a minor bradyarrhythmia characterized by paroxysmal 2:1 atrioventricular block.

### 3.2. Echocardiographic and Functional Data

#### 3.2.1. Systolic Function

For patients with functional single left ventricles, the mean EF was 58 ± 7%, with only three (8%) patients exhibiting an EF below 50%. Among those with a single right ventricle, the mean FAC was 40 ± 8%, with seven (23%) patients demonstrating a pathological FAC (below 35%). In two patients, evaluation of systolic function was not possible due to poor acoustic window. A significant correlation was observed among patients with a single right ventricle between pathological FAC and high NTpro-BNP values (Pearson’s r = −0.353, *p* = 0.071).

#### 3.2.2. Diastolic Function

Diastolic function was assessed in 63 patients (90%). The E/A ratio was pathological (E/A < 1) in four patients (6%), while the E/E’ ratio was pathological (E/E’ > 12) in eleven patients (17%). However, this percentage increased to 43% when using Margossian’s criteria, and further rose to 60% after the leg lifting test [16]. A competent or mild regurgitant atrioventricular valve was detected in 83% of the population (29 patients), while 17% exhibited at least moderate regurgitation of the atrioventricular valve or either valve (n = 6). Notably, five out of twenty-five patients (20%) with functional single right ventricles had more than moderate atrioventricular valve regurgitation. Echocardiographic findings are depicted in Table 2.

### 3.3. CPET Data

A total of 42 (60%) patients performed a cardiopulmonary exercise test. The main results are reported in Table 3. Mean VO_2_ max and the mean percentage of predicted VO_2_ max were, respectively, 26.4 ± 7.7 mL/Kg/min and 64.9 ± 13.9%. Mean ventilatory equivalent for carbon dioxide (Ve/VCO_2_) slope value was 33.7 ± 6. Peak Partial End-Tidal Carbon Dioxide (PETCO_2_) and the difference between basal and peak PETCO_2_ (delta PETCO_2_) were 32.1 ± 4.6 mmHg and 5.4 ± 3.4 mmHg, respectively. Mean Respiratory Exchange Ratio (RER) peak value was 1.4 ± 1.2.

Regarding the age of the patients, a mild inverse correlation was found with VO_2_ max resulting from the exercise test (Pearson’s ρ of −0.374, *p* = 0.035) and with oxygen saturation (Pearson’s ρ = −0.401, *p* = 0.001). However, no correlations were found between age and parameters of systolic function (EF, FAC) or diastolic function (E/E’, E/A). Nevertheless, age after Fontan completion presents a significant direct correlation with GLS (Pearson’s ρ = 0.371, *p* = 0.005), and IVC diameter (Pearson’s ρ = 0.493, *p* < 0.001). The latter was also inversely correlated with EF (Pearson’s ρ = −0.378, *p* = 0.018) and TAPSE (Pearson’s ρ = −0.808, *p* = 0.0019).

Among the 38 patients with left univentricular hearts, none of the patients exhibited at least moderate atrioventricular valve regurgitation, while among the 25 patients with right univentricular hearts, atrioventricular regurgitation was detected in five (20%) cases (*p* = 0.022). Furthermore, the time between birth and completion of Fontan palliation showed significant correlations with IVC diameter (Pearson’s ρ 0.423 and *p* < 0.01) and GLS value (Pearson’s ρ 0.430 and *p* = 0.001).

Functional VO_2_ max was inversely correlated with NTproBNP (Pearson’s ρ −0.301 with *p* = 0.025). It has also been shown that right systolic function expressed by quantification of FAC is significantly related to NTproBNP (Pearson’s ρ −0.353 with *p* = 0.071).

The GLS showed an inverse relationship with VO_2_ values measured during the cardiopulmonary stress test (Pearson’s ρ −0.475, *p* = 0.011) and a direct correlation with the time elapsed since the Fontan’s palliation (Pearson’s ρ 0.430, *p* = 0.001). Correlation analysis of echocardiographic and exercise findings is illustrated in Table 4.

### 3.4. Liver Assessment in Fontan Circulation

#### 3.4.1. Laboratory Exams

Regarding liver function, AST and ALT were normal in 87% and 90% of patients, respectively. GGT was pathological in 47 patients (68%) and there was a significant correlation between pathological values of GGT and the time elapsed since Fontan’s palliation (Pearson’s r 0.503, *p* < 0.001), whereas an inverse correlation with EF was documented (Pearson’s r −0.403, *p* = 0.011). Albumin and alpha-fetoprotein levels were found abnormal (<30 g/L, and ≥7.5 μg/L, respectively) in two (3%) and five patients (7%), respectively. Fecal alpha1antitrypsin was measured in 56 patients, and it was pathological (≥5 mg/dL) in 10 (18%). A total of 20 (29%) patients had thrombocytopenia, defined as platelet count <150,000/mm^3^.

#### 3.4.2. Fibroscan Data

**Figure 1:**
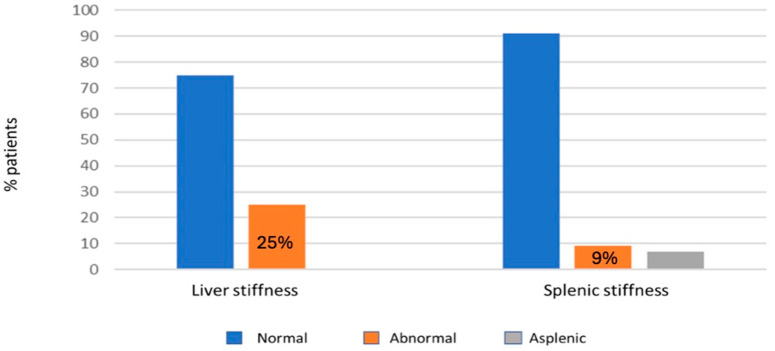
The distribution of liver and splenic stiffness in our cohort was assessed by transient elastography. The orange bars represent the percentage of patients with abnormal values in the assessment of liver and splenic stiffness in our cohort.

In 12 patients out of 60 (20%) an advanced stage of FALD was found, according to the criteria of Chemello et al. [13]. Fibroscan was performed on all patients. Liver stiffness was higher than the established cut-off value of 22 kPa in 17 patients (25%), with a mean value of 19 ± 9 kPa. For splenic stiffness, given the absence of a reference value for the Fontan population, a cutoff value of 46 kPa (derived from patients with non-FALD chronic liver disease) was established, revealing pathology in six out of sixty-five patients (9%) (Figure 1).

Among the 66 patients who underwent evaluation with abdominal ultrasound, blood chemistry tests, fibroscan, and hepatological visits, 36 patients (55%) showed no signs of progressive cardiogenic liver disease. Three scores were applied to assess the risk of evolution to advanced liver disease validated for patients with non-FALD chronic liver disease. LSPS score was calculated for 53 patients, of which 25 (47%) reported a pathological result. Regarding APRI score, it was altered in 27 patients (39%), while the FIB-4 showed the presence of advanced fibrosis (for values > 1.44) in six patients (9%) of which three (4%) were in an advanced stage of FALD (with FIB-4 values > 1.66). There was a strong correlation between hepatic stiffness and splenic stiffness (Pearson’s ρ 0.673 and *p* < 0.001) and an inverse relationship between platelet count and splenic stiffness (Pearson’s ρ −0.397 with *p* < 0.001). A higher prevalence of splenic stiffness alterations was also recorded in the right single ventricles compared to the left (*p* = 0.019). A significant association was found between the presence of atrioventricular valve regurgitation and liver stiffness (*p* = 0.015) (Table 5).

A positive correlation was found between time since cardiac surgery and age at admission with GGT (Pearson’s ρ 0.503 with *p* < 0.001 and 0.396 with *p* = 0.001, respectively). Statistically significant correlations were found between the IVC diameter, index of venous congestion, and the time elapsed since surgery with FIB-4 (Pearson’s ρ 0.369 and *p* = 0.002 and Pearson’s ρ 0.508 with *p* < 0.001, respectively). There was also a correlation between VO_2_ max and GGT (Pearson’s v −0.355 with *p* = 0.046) and VO_2_ max and AST (Pearson’s ρ −0.460 and *p* = 0.008) (Table 6).

## 4. Discussion

The enrolled population is composed of patients aged between 10 and 47 years at the time of admission, and the different effects of surgical palliation over time were evaluated. The majority had a single left ventricle, with extracardiac type conduit, since it has been demonstrated to reduce post-operative and long-term complications [1,24].

Most of the patients are in NYHA class I and II, considering that the average age of the studied population is 21 ± 9 years.

Tachyarrhythmias, particularly atrial tachycardias, are the most frequent type of arrhythmias in our population, as reported in the current literature [25,26]. Although most of the patients were in NYHA functional class I, NT pro-BNP was pathological in more than half of them and especially in those with systemic right ventricle (80% of them vs. 45% of those with left ventricle). This is probably because with the Fontan’s circulation, the morphologically right ventricle undergoes greater wall stress as compared to the left ventricle. To confirm this, a significant inverse correlation was observed between right systolic ventricular function and NT pro-BNP values.

In this study, few patients showed compromised ventricular function. However, EF was found to be strongly inversely correlated to GGT values. This data indicates how hepatic dysfunction, expressed by an increase in the GGT levels, is a consequence of a previous circulatory dysfunction, which can manifest itself with a reduced EF or a reduced exercise capacity. Age and time elapsed since Fontan’s surgery were significantly correlated with GGT, underlining a clear development of liver compromise over time due to chronic venous congestion.

Considering the diastolic function, only 18% of Fontan patients showed dysfunction and this percentage rises to 43% if the criteria of Margossian are considered; moreover, after the leg lifting test this percentage rises further to 60% [16]. The data agrees with the literature finding, considering both young and adult Fontan patients [27]. According to published papers, a relationship between the single right ventricle and diastolic dysfunction (expressed as an E/E’ ratio < 12) also emerged in our population with a progressive worsening over time [16,27].

In 83% of our population, the SAVV was continent or only mildly regurgitant. In this regard, a significant difference was found between the morphology of the univentricular heart (whatever right or left single ventricle) and the severity of the SAVVR: only one patient with a single left ventricle had moderate SAVVR, while 20% of those with right systemic ventricle had moderate-severe SAVVR. This is probably due to anatomical reasons: the right ventricle and the tricuspid annulus are not as performing as the systemic left ventricle and mitral valve in managing the systemic circulation.

When evaluating VO_2_ max in Fontan patients, it is also useful to note that VO_2_ max reflects both “central” cardiovascular factors, i.e., the transport of oxygen, and “peripheral” factors related to the skeletal muscle, i.e., extraction of oxygen [28,29,30]. In a cross-sectional study of 411 Fontan patients, it was demonstrated that central factors are responsible for about half of the physical and functional capacity of these patients: this is not surprising if we consider that we are dealing with a population that often has a lean body mass deficit and an associated deconditioning body [31].

According to the literature findings, in our population there are signs of hepatopathy linked to the univentricular circulation: this is documented by an increase in GGT greater than the hepatocytolysis indices, and from an instrumental point of view with a significant increase of hepatic stiffness compared to the cut-off used for non-Fontan patients diagnosed with liver disease (12 kPa). Using the cut-off proposed by the current literature for patients with univentricular circulation (22 kPa), the proportion of patients showing increased hepatic stiffness represents 25% of the total population. In a study of 129 Fontan patients undergoing hepatological evaluation with Fibroscan, this percentage is even higher, with about 70% of patients having values above the defined cut-off [13,16,27,31,32,33].

Furthermore, no patient showed protein synthesis deficiency, and no correlations emerged with the presence of symptoms associated with liver dysfunction data (GGT, liver stiffness, and NYHA class), underlining how Fontan-related liver disease has a clinically indolent course. However, the single ventricle ejection fraction was found to be strongly correlated with GGT values, as with VO_2_ max. This data shows how the hepatic dysfunction, expressed by increased GGT, is strictly linked to the functionality of the Fontan circulation. Patient’s age, time since surgery, and IVC diameter were significantly correlated with GGT, underscoring a clear development of liver disease over time due to chronic venous congestion.

In our study, the finding of atrioventricular valve regurgitation, at least moderate, was correlated to pathological hepatic stiffness and this might be due to volume overload which, reflecting upstream, causes greater venous congestion in the liver.

The execution of the Fibroscan also provided data on splenic stiffness. According to a study by the Italian group of Colecchia et al., splenic stiffness in non-FALD hepatic patients correlated better with portal hypertension severity than hepatic stiffness [34]. No significant correlations were found with other explored parameters (exercise test functional capacity, echocardiographic parameters). An indirect correlation between splenic stiffness values and thrombocytopenia was observed, since Fontan patients, having a higher incidence of hypersplenism and splenomegaly, suffer significant platelet sequestration. These data can be useful for assessment of FALD evolution over time.

Splenic stiffness has not been studied in the Fontan population yet. Although there are few studies on this topic, their findings and conclusions are conflicting and further research is needed for correct interpretation of this data in the population with univentricular physiology [35,36].

The presence of FALD was found to be correlated with Fontan’s age, considering the LSPS score: this datum is well known in the literature and intuitively linked to Fontan’s pathophysiology, while there does not seem to be a correlation between liver disease and morphology of ventricle.

A relationship between liver stiffness and functional capacity at CPET was also documented in our study [37]. Therefore, despite FALD having a clinically indolent course, the presence of a greater degree of hepatic venous congestion is the consequence of hemodynamic impairment of the univentricular circulation which reduces exercise VO_2_ max [38]. This observation highlights the role of measuring liver stiffness in Fontan patients as part of a multidisciplinary evaluation.

We also applied evaluation scores for non-FALD advanced liver disease in our population. One of these, the LSPS, according to the study by Chemello et al. on an adult Fontan population, appeared to have good sensitivity and specificity in the diagnosis of advanced FALD [13]. In our research, the validation of the cut-offs for advanced FALD with the cited scores (LSPS, APRI, FIB-4) was based on abdominal ultrasound (presence of two or more of the following parameters: splenic diameter >120 mm, portal vein > 12 mm, portal flow velocity < 12 cm/s or presence of collateral circulation on abdominal ultrasound evaluations).

In our population, the LSPS score was calculated for 53 patients, of which 47% reported a pathological result. The APRI score was altered in 39% of patients, while the FIB-4 showed the presence of advanced fibrosis (for values > 1.44) in 9% of patients, 4% of which are in an advanced stage of FALD (with FIB-4 values > 1.66). As far as the FIB-4 score is concerned, its correlation with the IVC diameter and with TAPSE demonstrate show venous congestion is an important determinant of liver damage in Fontan patients and how close the relationship between heart and liver function is. In our cohort, non-invasive FALD data were not validated by objective evidence on liver biopsy.

Surgical innovation and multidisciplinary management allowed children affected with CHD and univentricular physiology to survive into adulthood. Heart transplant is a feasible option which can be successful for many appropriately selected patients with failing Fontan circulation [39]. However, many controversies remain. Major concerns regard indications, benefits, and the optimal timing of transplantation.

At present there is not a uniform consensus to determine transplantation eligibility for patients with Fontan circulation [40]. With the onset of major Fontan-associated morbidities, the prognosis becomes more guarded. Renal dysfunction and hepatic dysfunction, which are common in long-term survivors, further modulate outcomes, but the impact of these factors is not fully understood. Consequently, more adult patients with Fontan are requiring combined heart-liver transplant.

Unfortunately, data delineating post-transplant outcomes and predictors for post-transplant mortality for the adult Fontan population remain limited [40].

## 5. Limitations

We acknowledge certain limitations in our study. This is a retrospective, single-institution cohort study with a low number of non-homogeneous enrolled patients and relative short follow-up. Secondly, transient elastography has not been designed for patients with central venous stasis and the evaluation of the severity of FALD based on this index is quite arbitrary, as it has not been validated by liver histology. However, we think that in Fontan patients FALD relies on the efficiency of the single ventricle circulation, and it reflects not only the entity of liver involvement but also the hemodynamic of the Fontan circuit.

This is a cross sectional study and provides the analysis of the CPET parameters and liver stiffness at single point; to better evaluate the possible correlations between exercise capacity and liver disease over time, prospective repeat measurement is necessary. The univentricular heart and Fontan palliation are rare conditions and in our study the cohort of enrolled patients is limited. Further studies should include more patients.

## 6. Conclusions

Our research confirms that Fontan physiology is an ongoing disease [41]. Cooperation between a multidisciplinary team, with careful periodic evaluation of cardiac and hepatic function, should be applied in the follow-up of all the Fontan patients from the beginning. The hepatic indexes, in particular GGT and liver transient elastography, strictly correlate with the hemodynamic condition of the Fontan circulation, and any increase in these indexes should encourage physicians towards a stricter Fcardiac evaluation.

SAVV dysfunction plays a major role in poorer functional outcomes and the progression of hepatic fibrosis, primarily due to increased venous congestion. It is crucial to establish a thorough multidisciplinary follow-up for these patients to identify complications early, provide timely treatment, and improve overall outcomes.

## Figures and Tables

**Table 1:** Demographics characteristics of our cohort. Complete atrioventricular canal (CAVC); Double-inlet left ventricle (DILV); Double-outlet right ventricle (DORV); Hemoglobin values sorted by sex (Hb, g/dL); Hypoplastic left heart syndrome (HLHS); Mitral atresia (MA); New York Heart Association functional class (NYHA); Pulmonary atresia and intact interventricular septum (PA/IVS); Single left ventricle (SLV); Systemic oxygen saturation after surgery (Sp02); Single right ventricle (SRV);Tricuspid atresia (TA);Total cavo-pulmonary connection (TCPC).

**Demographics**	**Fontan Population (n70)**
Age (years) on hospital admission (media ± SD)	21 ± 9
Male (*n*; %)	37 (52.9%)
Years after surgery (media ± SD)	16.4 ± 8.5
**Type of CHD (*n*; %)**	
TA	15 (21)
DORV	7 (10)
PA/IVS	8 (11)
HLHS	13 (19)
MA	4 (6)
DORV	3 (6.7)
CAVC unbalanced	5 (7)
DILV	11 (16)
Criss-cross heart	2 (3%)
Other	5 (7)
**Systemic ventricle physiology (*n*; %)**	
SLV	38 (54)
SRV	25 (36)
Indeterminate	7 (10)
**Type of surgery (*n*; %)**	
TCPC, intracardiac conduit	15 (21)
TCPC, external conduit	21 (49)
Bjork’s atrio-infundibular anastomoses	6 (9)
**Sp02 after surgery (media ± SD)**	92 ± 12.24
**NYHA class (*n*; %)**	
I	48 (72)
II-III	19 (28)
IV	0
**Biochemical data on admission** **(n;%); (media ± SD)**	
NT-pro-BNP > 125 ng/L	37 (55)
Hb (male population)	19.2 ± 10.7
Hb (female population)	14.4 ± 1.63
Lymphocytes (10^9^/L)	1.63 ± 1.4
**Treatment (*n*; %)**	
Warfarin	18 (26)
Cardioaspirin	42 (61)
Beta-Blockers	9 (13)
Vasodilators (*n*; %)	22 (32)

**Table 2:** Overview of echocardiographic results, with values and percentage/patients where applicable.

Parameter	Value	Patients, *n* (%)
Functional single left ventricle		
Mean EF	58 ± 7%	
EF < 50%		3 (8%)
Functional single right ventricle		
Mean FAC	40 ± 8%	
FAC < 35%		7 (23%)
Diastolic function		63 (90%)
Correlation FAC- NT-proBNP value	Pearson’s r = −0.353 (*p* 0.071)	
Pathological E/A ratio (E/A < 1)		4 (6%)
Pathological E/E’ ratio (E/E’ > 12)		11 (17%)
Pathological E/E’ ratio (Margossian’s criteria)		43%
Pathological E/E’ ratio (after leg lifting test)		60%
Atrioventricular valve regurgitation		
Competent or mild regurgitation		29 (83%)
Moderate or more regurgitation		6 (17%)
Functional single right ventricle with more than moderate regurgitation		5 (20%)

**Table 3:** Cardiopulmonary exercise test parameters in Fontan population (*n* = 42). (HR: heart rate; O_2_ oxygen; PETCO_2_: partial pressure end tidal carbon dioxide; RER: respiratory exchange ratio; SpO_2_: systemic oxygen saturation; VE/VCO_2_ ventilatory equivalent for carbon dioxide; VO_2_: oxygen consumption).

Parameter	Mean ± SD
VO_2_ peak	26.4 ± 7.7
VO_2_ % predicted	64.9 ± 13.9
O_2_ pulse rest	3.6 ± 1.1
O_2_ pulse	10.4 ± 2.7
Delta O_2_ pulse	6.9 ± 2.2
O_2_ pulse %	89.9 ± 15.8
VE/VCO_2_ slope	33.7 ± 6
SpO_2_ rest	95.5 ± 3.2
SpO_2_ peak	90.9 ± 4.2
Delta SpO_2_	−4.6 ± 2.5
RER peak	1.4 ± 1.2
HR max	151.7 ± 29.1
HR % predicted	78.7 ± 13.7
PETCO_2_ rest	26.6 ± 2.9
PETCO_2_ peak	32.1 ± 4.6
Delta PETCO_2_	5.4 ± 3.4

**Table 4:** Correlations between echocardiography and cardiopulmonary stress test and their corresponding Pearson’s correlation coefficients (ρ) along with *p*-values.

Parameter	Correlation Value	*p*-Value
Age		
VO_2_ max	Pearson’s ρ −0.374	0.035
Oxygen saturation	Pearson’s ρ −0.401	0.001
Systolic function (EF, FAC)	**-**	-
Diastolic function (E/E’, E/A)	**-**	-
GLS	Pearson’s ρ 0.371	0.005
IVC diameter	Pearson’s ρ 0.493	<0.001
IVC diameter		
EF	Pearson’s ρ −0.378	0.018
TAPSE	Pearson’s ρ −0.808	0.0019
Time between birth and Fontan Palliation		
IVC diameter	Pearson’s ρ 0.423	<0.01
GLS	Pearson’s ρ 0.430	0.001
Functional VO_2_ max		
NTproBNP	Pearson’s ρ −0.301	0.025
Right systolic function (FAC%)		
NTproBNP	Pearson’s ρ −0.353	0.071
GLS		
VO_2_ values	Pearson’s ρ −0.475	0.011
Time since Fontan palliation	Pearson’s ρ −0.430	0.001

**Table 5:** Relationship between degree of atrioventricular valve regurgitation and liver stiffness in our cohort.

Liver Stiffness (KPa)	Normal AV Valve or Mild Insuffiency (*n*,%)	≥ Moderate AV Valve Insufficiency (*n*,%)	Total Patients (*n*)
>22	14 (20%)	4 (6%)	18
<22	50 (71%)	2 (3%)	51

**Table 6:** Main findings related to hepatic function, risk scores, and functional parameters.

Parameter	Correlation Value (Pearson’s ρ)	*p*-Value
GGT		
Time since Fontan palliation	0.503	<0.001
EF%	−0.403	0.011
VO_2_ max	−0.355	0.046
Age at admission	0.396	0.001
AST		
VO_2_ max	−0.460	0.008
Splenic stiffness		
Hepatic stiffness	0.673	<0.001
Platelet count	−0.397	<0.001
FIB-4		
IVC diameter	0.369	0.002
Time since Fontan palliation	0.508	<0.001

## Data Availability

Data sharing is not applicable to this article.

## Abbreviations

SAVV	systemic atrio-ventricular valve
SAVVR	systemic atrio-ventricular valve regurgitation
TAPSE	tricuspid annular plane systolic excursion
MAPSE	mitral annular plane systolic excursion
EF	ejection fraction
FAC	fractional area change
GLS	global longitudinal strain
STE	speckle tracking echocardiography
NYHA	New York Heart Association
IVC	inferior vena cava
CPET	cardiopulmonary exercise test
VO_2_ max	maximum oxygen consumption
PLE	protein losing enteropathy
FALD	Fontan-associated liver disease
LSPS	liver stiffness-spleen size to platelet ratio
APRI	AST to platelet ratio index
FIB-4	Fibrosis 4
